# Involvement of miR-135a-5p Downregulation in Acute and Chronic Stress Response in the Prefrontal Cortex of Rats

**DOI:** 10.3390/ijms24021552

**Published:** 2023-01-13

**Authors:** Jessica Mingardi, Caterina Paoli, Luca La Via, Giulia Carini, Paulina Misztak, Carlo Cifani, Maurizio Popoli, Alessandro Barbon, Laura Musazzi

**Affiliations:** 1 Department of Medicine and Surgery, University of Milano-Bicocca, 20900 Monza, Italy; 2 Department of Molecular and Translational Medicine, University of Brescia, 25123 Brescia, Italy; 3 Pharmacology Unit, School of Pharmacy, University of Camerino, 62032 Camerino, Italy; 4 Department of Pharmaceutical Sciences, University of Milan, 20133 Milan, Italy

**Keywords:** stress, miR-135a-5p, prefrontal cortex, chronic mild stress, foot-shock stress, acute stress

## Abstract

Stress is a key risk factor in the onset of neuropsychiatric disorders. The study of the mechanisms underlying stress response is important to understand the etiopathogenetic mechanisms and identify new putative therapeutic targets. In this context, microRNAs (miRNAs) have emerged as key regulators of the complex patterns of gene/protein expression changes in the brain, where they have a crucial role in the regulation of neuroplasticity, neurogenesis, and neuronal differentiation. Among them, miR-135a-5p has been associated with stress response, synaptic plasticity, and the antidepressant effect in different brain areas. Here, we used acute unavoidable foot-shock stress (FS) and chronic mild stress (CMS) on male rats to study whether miR-135a-5p was involved in stress-induced changes in the prefrontal cortex (PFC). Both acute and chronic stress decreased miR-135a-5p levels in the PFC, although after CMS the reduction was induced only in animals vulnerable to CMS, according to a sucrose preference test. MiR-135a-5p downregulation in the primary neurons reduced dendritic spine density, while its overexpression exerted the opposite effect. Two bioinformatically predicted target genes, Kif5c and Cplx1/2, were increased in FS rats 24 h after stress. Altogether, we found that miR-135a-5p might play a role in stress response in PFC involving synaptic mechanisms.

## 1. Introduction

Behavioral stress is recognized as the major environmental predisposing and triggering factor for neuropsychiatric disorders including major depressive disorder (MDD) and post-traumatic stress disorder (PTSD) [1,2]. The hormonal, biochemical, and molecular changes that follow stress exposure, although adaptive in nature, can induce maladaptive consequences in vulnerable subjects, altering homeostasis and eventually precipitating the onset of pathological conditions [1,3]. The study of the mechanisms underlying these processes is relevant in the context of finding novel targets and pathways as putative, promising therapeutic tools for stress-related disorders.

Clinical evidence on stress-related psychiatric disorders highlighted volumetric reductions and synaptic dysfunction in corticolimbic areas (including the prefrontal cortex; PFC) [4]. At the same time, in animal models, stress was reported to induce dendritic retraction/spine loss in the same brain areas affected in patients, suggesting a role for dendritic and synaptic remodeling in etiopathogenesis [2,5,6,7]. Intriguingly, preclinical studies on rodents showed that even a single acute exposure to traumatic stress can induce not only rapid but also sustained changes in synaptic function (increase in glutamate release and transmission), neuroarchitecture (dendritic remodeling and reduction in synaptic spines), and behavior (impairment of mood and cognitive functions) [8,9,10].

Epigenetic modifications have been implicated in mechanisms regulating the brain response to stress [11]. Among those, miRNAs, small non-coding RNAs that fine-tune the expression of numerous genes in a rapid and reversible way, have been involved in the mechanisms of neuroplasticity, stress vulnerability, and pathophysiology in neuropsychiatric disorders [12,13,14,15,16,17,18,19]. Moreover, recent evidence highlighted a possible role of miRNAs as diagnostic biomarkers of stress-related disorders [20,21,22]. Among them, miR-135a-5p has been associated with stress response and dendritic spine remodeling as well as anxious-like behavior and the antidepressant effect [23,24,25]. Moreover, clinical studies showed a decrease in miR-135a levels in the blood of MDD patients and in the brain of suicide victims [23,26,27].

In the present work, we aimed to study whether miR-135a-5p plays a role in stress-induced changes in the PFC and to dissect the possible underlying mechanisms, comparing the effects of different validated stress models on male rats. PFC has been selected because having a key role in the top-down modulation of thought, action, and emotion has been implicated in the regulation of the stress response and the pathophysiology of stress-related psychiatric disorders [8,28,29,30]. As the acute, traumatic, and unpredictable stress, we applied foot-shock stress (FS), a protocol we previously reported to induce acute and long-lasting functional and morphological changes in the PFC [9,10], while chronic mild stress (CMS) was used as the classical model of depression [31]. We found that both acute and chronic stress decreased miR-135a-5p levels in the PFC of rats. Interestingly, in CMS rats, the reduction was selective for stress vulnerable (anhedonic) animals only. In vitro modulation of miR-135a-5p levels in primary neuronal cultures showed effects on spine remodeling. Bioinformatic analysis allowed for the identification of putative target genes involved in synaptic function, which were measured in the PFC of FS and CMS animals.

Our results suggest a central role for miR-135a-5p in the regulation of the synaptic processes induced by behavioral stress in the PFC.

## 2. Results

### 2.1. miR-135a-5p Levels Are Decreased by Both Acute and Chronic Stress in the Prefrontal Cortex of Rats

**Figure 1:**
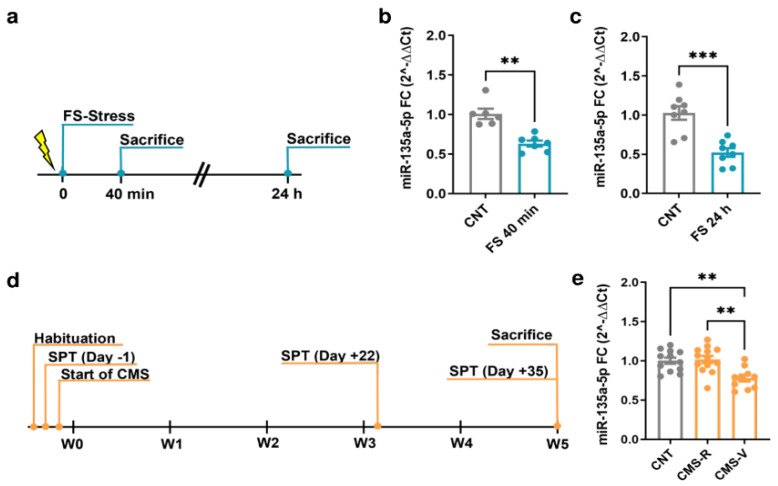
Changes of miR-135a-5p expression in the PFC of FS and CMS rats. (**a**) Experimental plan timeline of FS. Rats were subjected to a single session of FS and sacrificed immediately after 40 min or after 24 h. (**b**) miR-135a-5p expression levels measured by qPCR in the PFC of rats sacrificed immediately after stress. Mann–Whitney test, ** *p* < 0.01; N = 6 (CNT), 7 (FS). (**c**) miR-135a-5p expression levels measured by qPCR in the PFC of rats sacrificed 24 h after the beginning of stress. Student’s *t*-test, *** *p* < 0.001; N = 8 (CNT), 8 (FS). (**d**) Experimental plan timeline of CMS. Rats were subjected to 5 weeks of CMS and sacrificed after the last SPT on Day 35. (**e**) miR-135a-5p expression levels measured by qPCR in the PFC of rats. One-way ANOVA F = 2.374 (2, 23), Tukey’s post hoc test, ** *p* < 0.01; N = 12 (CNT), 13 (CMS-R), 10 (CMS-V).

MiR-135a-5p levels were measured in the PFC of FS and CMS rats using quantitative real-time PCR (Figure 1). Importantly, for CMS animals, we applied the sucrose preference test as a standard test for anhedonia, allowing to separate vulnerable (CMS-V) and resilient (CMS-R) rats depending on their anhedonic phenotype [19,32].

In the PFC of rats subjected to FS, a significant reduction in miR-135a-5p levels was found both immediately after the 40 min stress session (Mann–Whitney test; *p* < 0.01; Figure 1b) and 24 h after the beginning of stress (Student’s *t*-test; *p* < 0.001; Figure 1c). In CMS animals, miR-135a-5p levels were significantly reduced only in CMS-V rats compared to both the controls and CMS-R (one-way ANOVA, Tukey’s post hoc test: ** *p* < 0.01; Figure 1e), while in CMS-R rats the levels were not different from the controls (one-way ANOVA, Tukey’s post hoc test: *p* > 0.05 vs. CNT).

### 2.2. miR-135a-5p Modulation in Primary Cortical Neurons Alters Dendritic Spine Density and Dendritic Branching

**Figure 2:**
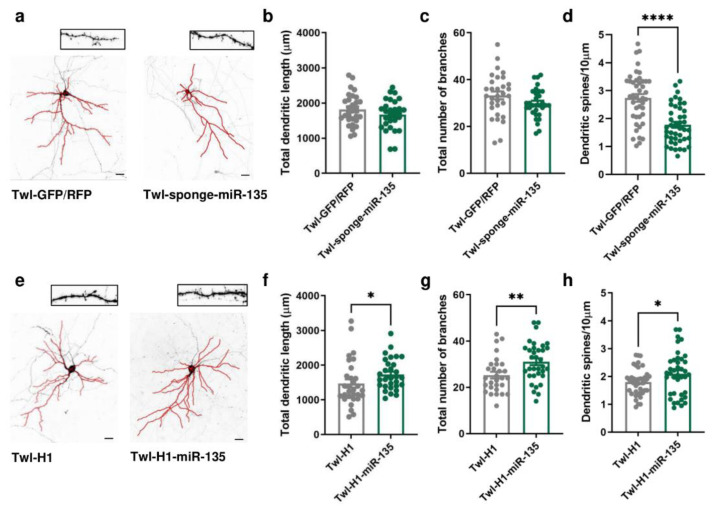
Morphological changes induced by in vitro miR-135a-5p modulation. (**a**) Representative reconstruction of neurons transfected with Twl-GFP/RFP (left) or Twl-sponge-miR-135 (right) and examples of dendritic spines that were analyzed. Scale bar 50 µm. (**b**) Total dendritic length, (**c**) total number of branches, and (**d**) dendritic spine density after downregulation of miR-135a-5p in pyramidal neurons. (**e**) Representative reconstruction of neurons transfected with Twl-H1 (left) or Twl-H1-miR-135 (right) and examples of dendritic spines that were analyzed. Scale bar 50 µm. (**f**) Total dendritic length, (**g**) total number of branches, and (**h**) dendritic spine density after over-expression of miR-135a-5p in pyramidal neurons. (**b**–**d**,**g**,**h**): Student’s *t*-test, * *p* < 0.05, ** *p* < 0.01, **** *p* < 0.0001; N = 30–40 (Twl-GFP/RFP), 30–40 (Twl-sponge-miR-135), 30–40 (Twl-H1), 30–40 (Twl-H1-miR-135). (**f**): Mann–Whitney test, * *p* < 0.05; N = 30 (Twl-H1), 30 (Twl-H1-miR-135).

To evaluate whether miR-135a-5p is directly involved in the neuronal morphological changes and dendritic spine remodeling of PFC pyramidal neurons, we transfected in DIV11 primary cortical neurons vectors specifically designed to overexpress or downregulate miR-135a-5p (Figure 2). Morphological analysis of pyramidal neurons 72 h post-transfection (DIV14) showed that miR-135a-5p downregulation did not affect the total dendritic length or number of branches (Figure 2b,c) but significantly decreased the dendritic spine density (Student’s *t*-test; *p* < 0.0001; Figure 2d). On the other hand, the over-expression of miR-135a-5p caused a significant increase in the total dendritic length (Mann–Whitney test, *p* < 0.05; Figure 2f), number of branches (Student’s *t*-test, *p* < 0.01; Figure 2g), and spine density (Student’s *t*-test, *p* < 0.05; Figure 2h).

### 2.3. Bioinformatic Analysis of miR-135a-5p Target Genes and In Vitro Validation of Selected Targets

**Figure 3:**
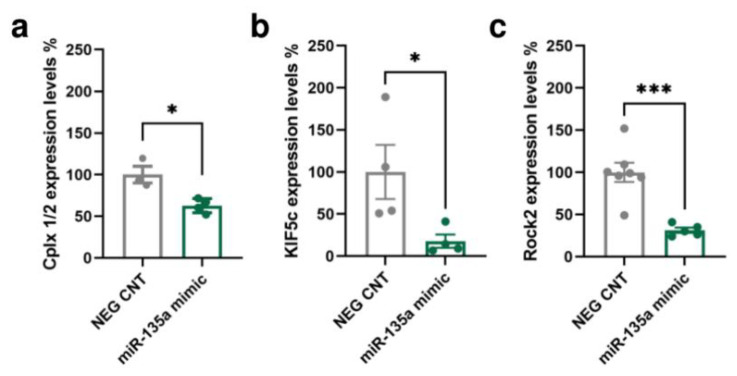
In vitro validation of predicted miR-135a-5p target genes. (**a**) Cplx 1/2, (**b**) Kif5c, and (**c**) Rock2 protein expression in primary neuronal cultures treated with miR-135a-5p mimics. (**a**,**b**) Mann–Whitney test, * *p* < 0.05; N = 4 (NEG CNT), 3–4 (miR-135a mimic). (**c**) Student’s *t*-test, *** *p* < 0.001; N = 7 (NEG CNT), 5 (miR-135a mimic).

A bioinformatic analysis of the miR-135a-5p target genes was conducted integrating the predictions of three algorithms: TargetScan [33], miRanda [34], and RNAhybrid [35]. The Multi Ontology Enrichment Tool [36] was used to highlight the “biological processes” most represented by the predicted target genes. Three genes consistently present within the most represented ontologies were selected: Cplx1/2 (“regulation of signaling”, “regulation of cell communication”, and “localization”), Kif5c (“anatomical structure morphogenesis” and “localization”), and Rock2 (“intracellular signal transduction”, “anatomical structure morphogenesis”, “regulation of signaling”, “regulation of cell communication”, and “localization”). Importantly, these genes have also been previously associated with neuronal remodeling, dendritic spine dynamics, and synaptic function (Cplx1/2 [37,38,39,40], Kif5c [41,42], and Rock2 [43,44,45]). The interaction of miR-135a-5p with the selected putative targets was evaluated in primary neuronal cultures transfected with specific mimics of miR-135a-5p (Figure 3). Western blot experiments showed that the expression of Cplx 1/2 (Mann–Whitney test, *p* < 0.05; Figure 3a), Kif5C (Mann–Whitney test; *p* < 0.05; Figure 3b), and Rock2 (Student’s *t*-test; *p* < 0.001; Figure 3c) was decreased in neurons treated with miR-135a-5p mimics. Overall, these data suggest that Cplx 1/2, Rock2, and Kif5c are validated biological targets of miR-135a-5p in cortical neurons.

### 2.4. Foot-Shock Stress Induces Time-Specific Changes in Kif5c and Cplx 1/2 Expression in the Prefrontal Cortex of Rats

**Figure 4:**
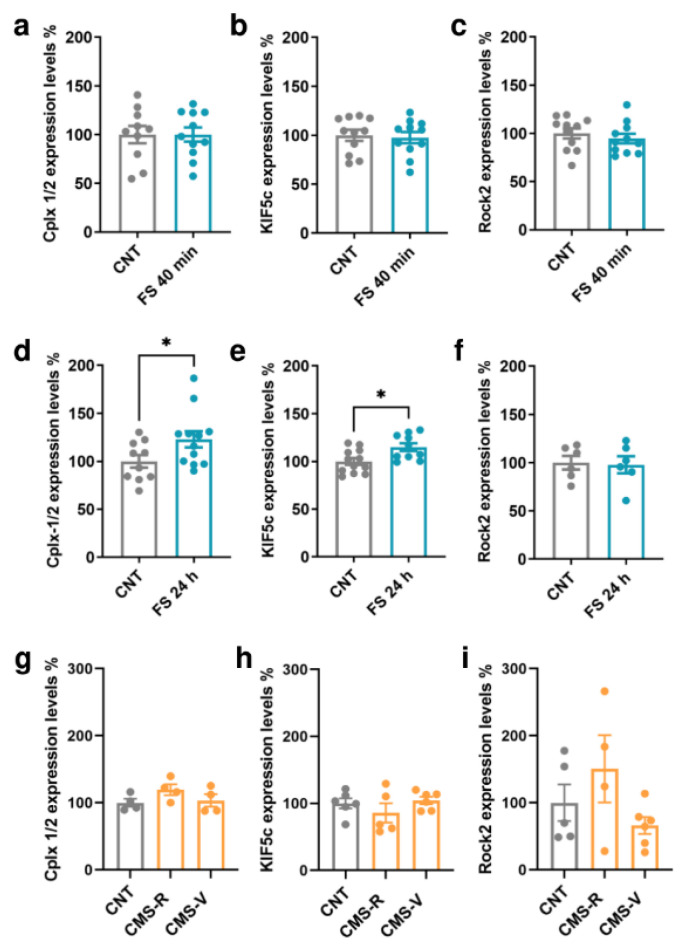
MiR-135a-5p target genes protein expression in the PFC of FS and CMS rats. (**a**) Cplx 1/2, (**b**) Kif5c, and (**c**) Rock2 protein expression in the PFC of FS rats immediately after stress. (**d**) Cplx 1/2, (**e**) Kif5c, and (**f**) Rock2 protein expression in the PFC of FS rats 24 h after the beginning of stress. Student’s *t*-test, * *p* < 0.05; N = 6–12 (CNT), 6–12 (FS). (**g**) Cplx 1/2, (**h**) Kif5c, and (**i**) Rock2 protein expression in the PFC of CMS rats. (**g**,**i**): Kruskal–Wallis test, Dunn’s post hoc test; N = 4–5 (CNT), 4 (CMS-R), 4–6 (CMS-V). (**h**): one-way ANOVA, Tukey’s post hoc test; N = 5 (CNT), 5 (CMS-R), 5 (CMS-V).

The protein expression of Cplx 1/2, Kif5c, and Rock2 was measured in the PFC of rats subjected to FS and CMS (Figure 4). In the PFC of FS rats, no significant changes were found in the expression of any of the targets when measured immediately after the acute stress session (Student’s *t*-test; *p* > 0.05; Figure 4a–c). However, when we looked at their expression 24 h after the beginning of stress, the levels of Kif5c and Cplx 1/2 were significantly increased in stressed rats compared to controls (Student’s *t*-test; Kif5c: *p* < 0.05, Cplx 1/2: *p* < 0.05; Figure 4d,e). No changes were found for Rock2 (Student’s *t*-test; *p*> 0.05; Figure 4f). On the other hand, in the PFC of CMS rats, we did not measure any significant change in the expression of the selected miR-135a-5p targets (Kif5c: one-way ANOVA; *p* > 0.05. Cplx 1/2, Rock2: Kruskal–Wallis test; *p* > 0.05; Figure 4g–i).

## 3. Discussion

In the present study, we collected evidence highlighting the involvement of miR-135a-5p in the response to both acute traumatic stress (FS) and CMS in the PFC of rats. Indeed, we measured reduced miR-135a-5p levels both immediately after FS exposure and in animals vulnerable to 5 weeks of CMS. A downregulation of miR-135a-5p in cortical pyramidal neurons in vitro was associated with a decreased number of dendritic spines and increased levels of Cplx1/2, Kif5c, and Rock2, which were validated as biological targets of miR-135a-5p in neurons. Cplx1/2 and Kif5c were also found to be increased in the PFC of FS rats 24 h after stress exposure.

In line with our findings, other studies reported decreased miR-135a-5p after exposure to different stress protocols. Repeated FS was found to reduce miR-135a-5p in the PFC of mice [24], while 2 h of restraint stress in mice reduced miR-135a in the amygdala [46]. Interestingly, in vivo knockdown of miR-135a in the basolateral amygdala increased anxiety-like behavior and amygdala spontaneous excitatory postsynaptic currents, suggesting increased glutamatergic neurotransmission [25].

On one hand, one single session of FS induced a rapid and long-lasting reduction in miR-135a-5p in the PFC of rats, already measurable immediately after the stress session and up to 24 h after. Using the same animal model, we previously demonstrated that the functional and dendritic remodeling effects of FS on the PFC are far from being only acute and may have an impact on behavior in the long term [8,9,10]. In more detail, we reported increased glutamate release and transmission in the PFC immediately after and at least up to 24 h after FS exposure [47,48,49], an increased number of excitatory synapses in only 40 min and of dendritic spines 24 h after stress [50,51], and a shortening and simplification of pyramidal neurons’ apical dendrites already measurable after 24 h and sustained for up to 2 weeks after stress [51,52]. Overall, the reduction in miR-135a-5p could represent one of the molecular underpinnings of both the acute and long-lasting consequences of FS stress, although in this model we cannot say whether this might be part of the adaptive or maladaptive response to stress. 

On the other hand, in the CMS protocol, the animals were phenotypically classified as resilient and vulnerable, and, intriguingly, miR-135a-5p levels were reduced only in CMS-V rats, suggesting a possible involvement of miR-135a-5p in the mechanisms of stress vulnerability/resilience. In line with this hypothesis, it has been previously shown that the downregulation of miR-135a-5p in raphe nuclei increased the anxiety-like behavior induced by chronic social defeat stress in mice, while miR-135a-5p overexpression increased resilience to stress and response to the antidepressant imipramine [23]. In previous studies, other miRNAs have been associated with stress vulnerability and depressive-like behaviors [12,13,14,15,16,17,18,19].

To understand the possible consequences of miR-135a-5p reduction on neuronal function, we focused on synaptic remodeling. Indeed, it was previously shown that miR-135a-5p supports long-lasting activity-dependent modifications of dendritic spines through mechanisms involving actin depolymerization and AMPA receptor exocytosis [37]. Using primary neuronal cultures, we found that miR-135a-5p downregulation decreased spine density of pyramidal neurons, while overexpression exerted the opposite effect, together with increasing neuronal arborisation and the dendritic length. Although these change results were obtained in primary neuronal cultures and were not confirmed in vivo, we might speculate that the reduction in miR-135a-5p in PFC could represent one of the mechanisms inducing synaptic and dendritic remodeling after stress exposure. Accordingly, alterations of neuronal architecture have been consistently proposed as key determinants of stress-induced psychopathology [2,5,6,7].

With the aim to unveil possible molecular mechanisms downstream miR-135a-5p downregulation, which could mediate spine remodeling under stress exposure, we ran a bioinformatic analysis and selected for validation three target genes involved in synaptic function and dendrite morphology: Cplx1/2, Kif5c, and Rock2. Cplx1/2 are presynaptic proteins regulating the stability of the soluble N-ethylmaleimide-sensitive factor attachment protein receptor (SNARE) complex, with both inhibitory and facilitatory functions in the fusion of vesicles [53,54]. In a previous study, the regulation of Cplx1/2 induced by miR-135a-5p was involved in long-lasting spine remodeling induced by long-term depression (LTD) in cortical neurons [37]. Kif5c is a member of the kinesin family involved in the transport of RNA substrates for local translation and has never been validated before as a target of miR-135a-5p. Alterations of Kif5c expression were linked to impairments in structural plasticity and memory [41,55]. Rock2, a serine/threonine kinase, takes part in actin cytoskeleton organization, and its deletion has been linked to anxiety-like behavior and the alteration of dendritic spine density and morphology in the hippocampus [56,57,58]. To the best of our knowledge, we give the first demonstration of Kif5c as a validated target gene of miR-135a-5p here.

Measuring the three validated target in PFC samples from FS and CMS rats, we observed that protein expressions of Cplx1/2 and Kif5c were significantly increased 24 h after FS exposure (a time point in which miR-135a-5p was still decreased). The lack of changes in the Cplx1/2 and Kif5c levels immediately after FS is not surprising, because it is possible that, although the decrease in miR-135a-5p occurs very rapidly in stress conditions, a longer time is required for the disinhibition of the translation of target proteins. Differently, no changes in the expression of Kif5c, Cplx1/2, and Rock2 were detected in CMS rats. Considering that the CMS protocol runs for over 5 weeks, during which molecular and phenotypical changes are shaped on adaptive mechanisms, it could be speculated that Kif5c and Cplx1/2 may play a role in the synaptic remodeling induced by stress in early phases, while in the long term other mechanisms could be involved, and Kif5c and Cplx1/2 expression is restored. Moreover, other target genes could be implicated in the functional and morphological effects of both acute and chronic stress as well. Further studies are needed to elucidate the biological pathways downstream miR-135a-5p that could contribute to shaping the stress response in PFC.

## 4. Materials and Methods

### 4.1. Animals

Experiments were performed in accordance with the European Community Council Directive 2010/63/UE and were approved by the Italian legislation on animal experimentation (Decreto Legislativo 26/2014, authorizations N 308/2015-PR and 521/2015-PR). Adult Sprague–Dawley male rats were used in FS experiments (350–450 g, Charles River, Calco, Italy). For CUMS experiments, the rats used were 175–200 g in weight at the beginning of the 5 weeks of protocol (350–450 g at the end). Rats were housed two per cage at 20–22 °C, in 12 h light/dark cycle (light on 7:00 a.m. off 7:00 p.m.), with water and food ad libitum, except when required for CMS or FS.

### 4.2. Chronic Mild Stress (CMS) Paradigm

The rats belonging to the CMS group were exposed once or twice daily to random, mild, and unpredictable stressors for five weeks, as previously reported [19,32]. The stressors included in the protocol were as follows: food/water deprivation (8–12 h), overcrowding (rats were randomly housed five per cage for 6–12 h), isolation (each rat was individually housed for 6–12 h), soiled cage (rats were housed in cages soiled with 500 mL of water in the sawdust for 6–12 h), cage tilting (cages were tilted 45° left or right for 6–12 h), light on overnight, light/dark reversal, and forced swim (rats were forced to swim in 40 cm of room temperature water in a 30 cm in diameter Plexiglas cylinder, for 5 min once a week). Stress was applied at different times of the day and with a different length of time, in order to minimize prediction. CNT rats were left undisturbed in their home cages, except for sucrose preference test and weight measurement (twice a week).

### 4.3. Sucrose Preference Test

Sucrose preference test was used to assess anhedonic phenotype and to divide stressed rats in CMS resilient (CMS-R) and vulnerable (CMS-V), applying a cut-off of preference at 55%, as previously reported [19,32].

### 4.4. Foot-Shock Stress (FS) Paradigm

Rats were subjected to a single session of acute inescapable FS stress, as previously reported [52,59]: intermittent shocks (0.8 mA) for 40 min (20 min total of actual shock with random intershock length between 2 s and 8 s). The FS box was connected to a scrambler controller (LE 100-26, Panlab, Barcelona, Spain) that delivered intermittent shocks to the metallic floor. Control animals were left undisturbed in their home cages.

### 4.5. Primary Cortical Neuronal Cultures

Primary cortical neurons were prepared as described before [52]. Briefly, cortices from mice embryos at embryonic day 16.5 were dissociated mechanically, and neurons were resuspended in Neurobasal™ medium supplemented with B27 (Gibco™, Thermo Fisher Scientific, Waltham, MA, USA) containing 30 U/mL Penicillin, 30 mg/mL Streptomycin (Sigma-Aldrich, Milan, Italy), 0.75 mM Glutamax (Gibco™, Thermo Fisher Scientific), and 0.75 mM L-Glutammine (Gibco™, Thermo Fisher Scientific). Depending on the experiment, neurons were seeded 80.000 cells/1.9 cm^2^ on 0.1 mg/mL Poly-D-Lysine-coated glass coverslip (Sigma-Aldrich, for transfection with miRNA expression vectors) or 300.000 cells/10 cm^2^ on 0.02 mg/mL Poly-D-Lysine-coated 6-multiwell plates (for transfection with miRNA mimics) and maintained at 37 °C under a 5% CO_2_ humid atmosphere. Three days after seeding, half of the medium was replaced with 24 h astrocyte-conditioned medium. Then, half of the medium was changed every seven days for up to a maximum of four weeks.

### 4.6. RNA Isolation, Reverse Transcription, and Real-Time PCR

Total RNA was extracted from rat PFC and primary neuronal cultures using Tri-Reagent (Sigma-Aldrich) and Direct-zol RNA MiniPrep (Zymo Research, Freiburg, Germany), in accordance with the instructions of the manufacturer [19,32]. Reverse transcription was carried out using miRCURY^®^ LNA^®^ RT kit (Exiqon, QIAGEN, Milano, Italy). qPCR was performed using iTaq Universal SYBR Green supermix (Bio-Rad Laboratories, Milano, Italy). Primers used for qPCR are as follows: miR-135a-5p, YP00204762; SNORD68, YP00203911; RNU1A1, and YP00203909 (miRCURY^®^ LNA^®^ miRNA PCR assay, Exiqon). The relative expression of miRNAs was calculated using the comparative Ct (ΔΔCt) method and is expressed as fold change. The mean of SNORD68 and RNU1A1 was used as a control reference.

### 4.7. DNA Constructs

Vectors to overexpress and downregulate miR-135a-5p were designed starting from Twl-PGKgfp-H1 (Twl-H1) and Twl-PGKgfp-CMVrfp (Twl-GFP/RFP) vectors, respectively [19]. To obtain the overexpression of miR-135a-5p, specific oligonucleotides were designed on the sequence of miR-135a-5p and annealed in vitro to produce a dsRNA (ds-miR-135a-5p; TOP: CTAGAGTATGGCTTTTTATTCCTATGTGACTGCAGTCACATAGGAATAAAAAGCCATATTTTTTC, BOTTOM: TCGAGAAAAAATATGGCTTTTTATTCCTATGTGACTGCAGTCACATAGGAATAAAAAGCCATACT). After digestion with XhoI and XbaI (Thermo Fisher Scientific), ds-miR-135a-5p oligonucleotides were cloned in Twl-H1 using T4 DNA Ligase (Thermo Fisher Scientific). The vector obtained was Twl-PGKgfp-H1-miR-135a-5p (Twl-H1-miR-135). The downregulation vector was generated by cloning a sequence containing 3 repeats of a complementary sequence to miR-135a-5p interspersed with spacer nucleotides, acting as a sponge for endogenous miR-135a-5p (ds-sponge-miR-135a-5p; TOP: TCGACTCACATAGGAATAAAAAGCCATATGACTGCAGCGTTCACATAGGAATAAAAAGCCATATGAGTCAGTCATTCACATAGGAATAAAAAGCCATAG, BOTTOM: TCGACTATGGCTTTTTATTCCTATGTGAATGACTGACTCATATGGCTTTTTATTCCTATGTGAACGCTGCAGTCATATGGCTTTTTATTCCTATGTGAG) [60]. Ds-sponge-miR-135a-5p was inserted within the 3′UTR of GFP, digesting Twl-GFP/RFP with SalI (Thermo Fisher Scientific). The vector obtained was Twl-PGKgfp-CMVrfp-sponge-miR-135a-5p (Twl-sponge-miR-135). RFP expression was used as a control for transfection, while GFP expression was dependent on the presence of endogenous miR-135a-5p binding to the sponge. The empty vectors Twl-H1 and Twl-GFP/RFP were used as controls.

### 4.8. Transfection of Neuronal Cultures

Neuronal cultures were transfected at DIV11 (miRNA expression vectors; miR-135a-5p miRCURY LNA miRNA mimic, 5′-UAUGGCUUUUUAUUCCUAUGUGA-3′ ID: YM00473104, Exiqon; negative control 5′-TAACACGTCTATACGCCCA-3′ ID: YI00199006, Exiqon) using Magnetofection™ Technology (Oz Biosciences, Marseille, France), in accordance with the protocol of the manufacturer [19]. Briefly, the vectors (miRNA expression vectors) and miR-135a-5p LNA mimic/negative control were incubated with NeuroMag magnetic beads (Oz Biosciences) in the ratio of 1 μg DNA:3.5 μL NeuroMag or 5–25 pmol LNA/RNA:3.5 μL NeuroMag, respectively, to 100 μL of Neurobasal free medium for 20 min and added drop-by-drop to the cells. Then, the cells were incubated on a magnetic plate for 15 min at 37 °C. After 1 h, a complete change of medium was performed, and cells were kept at 37 °C under a 5% CO_2_ humid atmosphere. Then, 72 h post-transfection, neuronal cells were fixed in 4% PFA for 20 min at room temperature and mounted on coverslip using SlowFade™ Diamond Antifade Mountant (Thermo Fisher Scientific) for confocal imaging (miRNA expression vectors), or mechanically harvested in 100 µL of RIPA for protein purification (miR-135a-5p LNA miRNA mimic/negative control).

### 4.9. Confocal Microscopy and Imaging Analysis

Images of fluorescently labeled cells were taken using a confocal microscope LSM880 Upright (Carl Zeiss, Jena, Germany) with a 20× objective at a resolution of 1980 (x/y) pixels. Pictures represent maximum intensity projections of 5 consecutive optical sections taken at an 8 µm interval. Total dendritic length and number of branches were manually traced using Simple Neurite Tracer from Fiji (NIH, Bathesda, MD, USA) [61]. For each condition, a minimum of 30 cells was analyzed. The number of spines was measured manually using ImageJ (NIH, Bathesda, MD, USA), and spine density was calculated by quantifying the number of spines in a 10 µm dendritic segment. For each condition, spines of three secondary dendrites from a minimum of 15 cells were analyzed.

### 4.10. Bioinformatic Prediction of miR-135a-5p Target Genes

MiR-135a-5p target prediction was evaluated using the combination of three different algorithms: TargetScan [33], miRanda [34], and RNAhybrid [35]. The Multi Ontology Enrichment Tool [36] was queried to investigate the most represented pathways involving miR-135a-5p target genes. Selected targets were: Complexin 1/2 (Cplx1/2), Kinesin family member 5C (Kif5c), and Rho Associated Coiled-Coil Containing Protein Kinase 2 (Rock2).

### 4.11. Western-Blotting

BCA protein concentration assay (Merk Life Science S.r.l., Milano, Italy) was used for protein quantification. Western blotting was performed as previously described [49,62]. Primary antibodies used were: Complexin 1/2 (1:1000, cod. 122002, Synaptic System, Goettingen, Germany), Kif5c (1:1000, cod. Ab193352, Abcam, Cambridge, UK), and Rock2 (1:1000, cod. 04-841, Merk Life Science). Antibodies against GAPDH (1:6000, cod. Mab374, Merk Life Science) or α-Tubulin (1:20,000, cod. ab7291, Abcam) were used as internal controls. Luminescence or chemiluminescence were used for signal detection. Secondary antibodies used were anti-mouse and anti-rabbit horseradish peroxidase (HRP)-conjugated (1:2000, Merk Life Science) or fluorophor-conjugated antibodies (1:2000, IRDye 800CW goat anti-rabbit IgG 926-32211 or IRDye 680RD goat anti-mouse IgG 926-68020, LI-COR, Bad Homburg, Germany). Chemiluminescent signals were detected using an enhanced chemiluminescence (ECL) kit (GE Healthcare Life Sciences, Milan, Italy) and visualized with Chemidoc XRS (Bio-Rad Laboratories). Fluorescent signals were detected using an Odyssey infrared imaging system (LI-COR Biosciences) and analyzed using Odyssey version 1.1 (LI-COR Biosciences). Image Studio software (LI-COR Biosciences) was used for quantification.

### 4.12. Statistical Analysis

Data were shown as mean ± standard error of mean (SEM). Statistical analysis of the data was performed using GraphPad Prism 8 (GraphPad Software Inc., San Diego, CA, USA). Normal distribution was verified using Kolmogorov–Smirnov test. For normally distributed data, statistical analyses were performed with unpaired Student′s *t*-test or one-way analysis of variance (ANOVA) when appropriate, followed by Tukey’s post hoc multiple comparison test. For non-normally distributed data, statistical analyses were performed with the Mann–Whitney test (when 2 groups were compared) or Kruskal–Wallis test, followed by Dunn′s multiple comparison test. Statistical significance was assumed at *p* < 0.05.

## 5. Conclusions

In this work, we showed that both acute and chronic stress induced in the PFC of rats caused a downregulation of miR-135a-5p. MiR-135a-5p modulation in primary neuronal cultures caused a remarkable remodeling of dendritic spines and was used to validate Cplx1/2, Kif5c, and Rock2 as biological target genes in neurons.

Although other miRNAs have been implicated in the stress response and in stress-dependent dendritic remodeling [12,13,14,63,64], our results support a central role for miR-135a-5p, so downregulation could be proposed as an early and long-lasting hallmark of stress in the PFC.

## Data Availability

Not applicable.
